# Small Bowel Obstruction due to Anomalous Congenital Bands in Children

**DOI:** 10.1155/2016/7364329

**Published:** 2016-07-10

**Authors:** Basak Erginel, Feryal Gun Soysal, Huseyin Ozbey, Erbug Keskin, Alaattin Celik, Aslı Karadag, Tansu Salman

**Affiliations:** Department of Pediatric Surgery, Istanbul Medical Faculty, Istanbul University, 34093 Istanbul, Turkey

## Abstract

*Introduction*. The aim of the study was to evaluate our children who are operated on for anomalous congenital band while increasing the awareness of this rare reason of intestinal obstruction in children which causes a diagnostic challenge.* Patients and Methods*. We retrospectively reviewed the records of fourteen children treated surgically for intestinal obstructions caused by anomalous congenital bands.* Results*. The bands were located between the following regions: the ascending colon and the mesentery of the terminal ileum in 4 patients, the jejunum and mesentery of the terminal ileum in 3 patients, the ileum and mesentery of the terminal ileum in 2 patients, the ligament of Treitz and mesentery of the jejunum in one patient, the ligament of Treitz and mesentery of the terminal ileum in one patient, duodenum and duodenum in one patient, the ileum and mesentery of the ileum in one patient, the jejunum and mesentery of the jejunum in one patient, and Meckel's diverticulum and its ileal mesentery in one patient. Band excision was adequate in all of the patients except the two who received resection anastomosis for intestinal necrosis.* Conclusion*. Although congenital anomalous bands are rare, they should be considered in the differential diagnosis of patients with an intestinal obstruction.

## 1. Introduction

Intestinal bands caused by inflammation and surgery in pediatric patients are common and can lead to intestinal obstruction. However, anomalous congenital bands that are not related to abdominal conditions such as laparotomy, trauma, or peritonitis are extremely rare causes of intestinal obstruction in children [1]. Their exact incidence is unknown [2]. The etiology of anomalous congenital bands is still unknown, but they are not secondary to known embryologic remnants such as omphalomesenteric duct or vitelline vessel remnants [3]. The importance of anomalous congenital bands is related to the difficulty of diagnosis, and fatal cases have been reported with late diagnosis [4]. Here, we report fourteen children with congenital anomalous bands, which represent the largest series reported in the literature as far as we investigated. The aim of our study was to increase the awareness of this rare condition in children admitted with intestinal obstruction.

## 2. Methods

During the twenty-five-year period from 1990 to 2015, there were 14 children treated surgically for congenital anomalous bands in Department of Pediatric Surgery, Istanbul Medical Faculty, Istanbul University. All of the patients were admitted with clinical diagnosis of acute intestinal obstruction. Annual distribution of patients was even. Almost every year we had one patient. The cases are retrospectively evaluated for demographic findings, clinical signs, diagnostic methods, preoperative findings, band location, and postoperative complications.

## 3. Results

**Figure 1:**
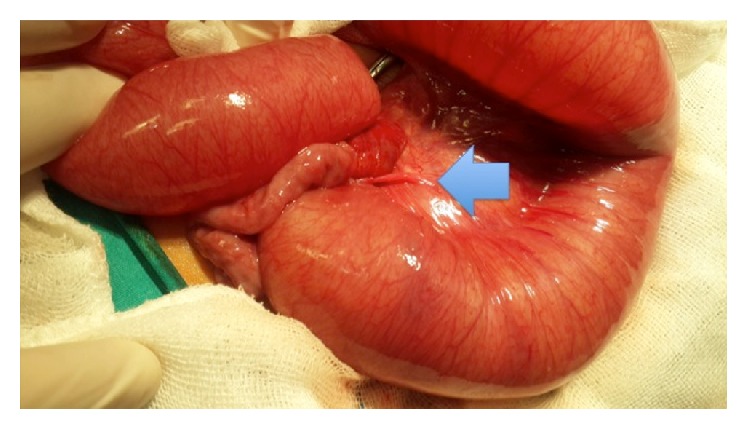
A congenital band between the ileum and mesentery of the terminal ileum.

There were 4 girls and 10 boys ranging in age between 4 days and 12 years (mean age: 6.6 ± 3.2 years). The two newborn patients were presented with abdominal distention and vomiting. The remaining 12 patients had abdominal pain and vomiting as the presenting symptom. There were two patients that complained of chronic abdominal pain. There was no history of prior abdominal surgery in any of the patients. The blood tests and electrolyte levels were examined in all patients. A plain abdominal graphy revealed air-fluid levels in all patients. Our routine management of suspected acute abdomen and intestinal obstruction includes an abdominal ultrasonography. The ultrasonography revealed dilated intestinal loops in all patients. There were four patients with a partial intestinal volvulus. An abdominal computed tomography (CT) was performed in two of the patients who had complained of chronic abdominal pain. In those patients, the CT revealed dilatation of the small bowel segments. Intravenous fluids were promptly administered, and nasogastric tubes were placed. Antibiotic treatment is routinely started in all patients with acute intestinal obstructions. An open laparotomy identified a congenital anomalous band in all of the patients.  Figure 1 presents an anomalous congenital band between the ileum and mesentery of the terminal ileum. The anatomic localisations of the bands are given in Table 1.

The well-vascularized bands were ligated and divided in all of the cases. Two patients with intestinal necrosis due to compression of the band were treated with intestinal resection and anastomosis. In the patient in whom the band was between Meckel's diverticulum and its ileal mesentery, wedge resection was performed besides the band excision.

The mechanism of intestinal obstruction in the patients is given in Table 2.

The histopathological examination of all the patients revealed an anomalous congenital band with loose connective tissue containing blood vessels and nerves.

## 4. Discussion

Anomalous congenital bands are rare causes of intestinal obstructions in children. The etiology of anomalous congenital bands has not been elucidated. Anomalous congenital bands were first defined by Touloukian [5]. There are reports of intestinal obstruction due to congenital bands without any evidence of inflammation or trauma. The bands are not frequently encountered and are difficult to diagnose and classify. There are a limited number of case reports and a few series [1, 6]. The largest series was reported by Akgür et al. [6] and included 8 children with anomalous congenital bands. This study is the largest case series with 14 children in the English literature. In the series of Akgür et al. [6], the most common band location was between the ascending colon and the terminal ileum in 4 of 8 patients (50%). In our series, the most common location was also the ascending colon and the mesentery of the terminal ileum. We speculate that the ascending colon is the most common location of an anomalous congenital band. Their second most common location was between the ligament of Treitz and the mesentery of the terminal ileum. This location was observed in 2 of 8 patients (25%) [6]. We also experienced 1 case with a band located between the ligament of Treitz and the mesentery of the terminal ileum. In two of their patients (2/8), the bands originated from the liver and were attached to the mesentery. However, there were no cases in our study with liver attachments.

An intestinal obstruction is caused by one of three mechanisms: compression of the bowel, partial volvulus, or entrapment of an intestinal loop between the band and mesentery. In our study, the obstructive mechanisms were bowel compression by a band in eight patients (57.1%), partial volvulus in four patients (28.6%), and entrapment of an intestinal loop between the band and mesentery in two patients (14.3%). The obstructive mechanisms were compression of bowel by the band in five patients (62.5%) and entrapment of an intestinal loop between the band and mesentery in three patients (37.5%) in the series by Akgür et al. [6]. In both series, the most common reason for the obstruction was bowel compression by a band.

Anomalous congenital bands do not arise from the described embryonic structures. Therefore, the location varies in every patient. Several anatomical locations have been described in case reports. Maeda et al. [7] reported a 17-year-old boy with an anomalous congenital band extending from the antimesenterium of the terminal ileum to the mesoappendix. Liu et al. [8] reported a patient with a congenital band extending from the antimesenteric wall of the proximal jejunum to the ligament of Treitz, which was the second case in the literature of an anomalous congenital band causing proximal jejunal obstruction.

Anomalous congenital bands can occur in multiple locations within the same patient [9]. One of our patients was a three-year-old girl with two congenital bands. One of the bands extended from the antimesenterium to the mesenterium of the jejunum, and the other band was located between the antimesenterium and the mesenterium of the ileum. Kostic et al. [10] also reported two bands in a single patient.

All of our patients were admitted with findings of acute intestinal obstruction. In newborn patients, the presence of abdominal distension is the cardinal sign because abdominal pain cannot be detected. However, the major finding is abdominal pain and vomiting in infants and elderly patients.

The band was excised in all 12 patients, and the surgical treatment was adequate. However, there were two patients with intestinal necrosis due to compression of the band. These patients were treated with intestinal resection and anastomosis.

The bands can occur in various anatomical locations and cause a spectrum of clinical pathologies including sigmoid volvulus and internal hernia [11, 12].

Although laparotomy and band excision are the treatment of choice in patients with congenital band, successful results with laparoscopic excisions have also been reported [13]. A limitation of our study is the lack of laparoscopic band excision.

This is currently the largest series of congenital anomalous bands in children. Although congenital anomalous bands are rare, they should be considered in the differential diagnosis of patients with intestinal obstructions.

## Figures and Tables

**Table 1:** The location of the congenital band and the surgery type.

	Age	Gender	Localization (origin-attached mesentery)	Operation
1	4 d	M	T. ileum-ascendant colon	Band excision
2	1 m	F	T. ileum-ileum	Band excision + ileal resection-anastomosis
3	3 y	F	Jejunum-jejunum ileum-ileum	Band excision
4	3.5 y	M	Ascendant colon-terminal ileum	Band excision
5	4 y	M	Ascendant colon-terminal ileum	Band excision
6	5 y	M	Jejunum-terminal ileum	Band excision
7	5 y	F	Ascendant colon-terminal ileum	Band excision + ileal resection-anastomosis
8	5 y	M	Ileum-terminal ileum	Band excision
9	6 y	M	Treitz-jejunum	Band excision
10	10 y	F	Jejunum-terminal ileum	Band excision
11	10 y	M	Meckel's diverticulum-ileum	Band excision-wedge resection
12	10 y	M	Treitz-terminal ileum	Band excision
13	10 y	M	Duodenum-duodenum	Band excision
14	12 y	M	Jejunum-terminal ileum	Band excision

d: days; m: months; y: years.

**Table 2:** The mechanism of intestinal obstructions.

Mechanism	Number of patients
Band compression	8
Segmental volvulus	4
Entrapment of an intestinal loop	2
